# A computational method for prediction of xylanase enzymes activity in strains of *Bacillus subtilis* based on pseudo amino acid composition features

**DOI:** 10.1371/journal.pone.0205796

**Published:** 2018-10-22

**Authors:** Shohreh Ariaeenejad, Maryam Mousivand, Parinaz Moradi Dezfouli, Maryam Hashemi, Kaveh Kavousi, Ghasem Hosseini Salekdeh

**Affiliations:** 1 Department of Systems Biology, Agricultural Biotechnology Research Institute of Iran (ABRII), Agricultural Research Education and Extension Organization (AREO), Karaj, Iran; 2 Department of Microbial Biotechnology, Agricultural Biotechnology Research Institute of Iran (ABRII), Agricultural Research Education and Extension Organization (AREO), Karaj, Iran; 3 Institute of Biochemistry and Biophysics (IBB), University of Tehran, Tehran, Iran; National Chiao Tung University College of Biological Science and Technology, TAIWAN

## Abstract

Xylanases are hydrolytic enzymes which based on physicochemical properties, structure, mode of action and substrate specificities are classified into various glycoside hydrolase (GH) families. The purpose of this study is to show that the activity of the members of the xylanase family in the specified pH and temperature conditions can be computationally predicted. The proposed computational regression model was trained and tested with the Pseudo Amino Acid Composition (PseAAC) features extracted solely from the amino acid sequences of enzymes. The xylanases with experimentally determined activities were used as the training dataset to adjust the model parameters. To develop the model, 41 strains of *Bacillus subtilis* isolated from field soil were screened. From them, 28 strains with the highest halo diameter were selected for further studies. The performance of the model for prediction of xylanase activity was evaluated in three different temperature and pH conditions using stratified cross-validation and jackknife methods. The trained model can be used for determining the activity of newly found xylanases in the specified condition. Such computational models help to scale down the experimental costs and save time by identifying enzymes with appropriate activity for scientific and industrial usage. Our methodology for activity prediction of xylanase enzymes can be potentially applied to the members of the other enzyme families. The availability of sufficient experimental data in specified pH and temperature conditions is a prerequisite for training the learning model and to achieve high accuracy.

## Introduction

After cellulose, xylan is the second most abundant polysaccharide in nature which is mostly found in the plant cell wall and accounts for a large part of plants biomass. Xylan can be depolymerized using xylanase enzymes, an important family of hydrolases.

The glycosyl hydrolases (GHs) are a very large family of enzymes which hydrolyze the glycosidic bond between carbohydrates as well as between a carbohydrate and a noncarbohydrate moiety to form heteropolysaccharides. The classification of GH enzymes into subfamilies is mainly based on amino-acid sequence similarities as proposed in [1–7].

Endo-xylanases with somewhat different sequences are found in various GH families because of the sequence-based classification of GH enzymes and despite similar structures and conserved folding [3–9].

Xylanases (EC3.2.1.X) are among important constituent subfamilies of GH enzymes. While xylanase isoenzymes show different specificities, they have synergistic effect on the hydrolysis of xylan [10]. Heteroxylan backbone is composed of glycoside linkages. For cleaving these bonds, the interaction of some cleavage enzymes for both main and side chains is required.

Endo-β-1,4-xylanases (EC 3.2.1.8), β-1,4 xylosidases (EC 3.2.1.37), and exoxylanases are examples of enzymes with the capability of cleaving main-chain glycosyl groups [7]. Most xylanases extracted from microbial communities are single-subunit enzymes [9].

There have been a lot of works to achieve highly active xylanases suitable for various applications in specified conditions [11–13]. A comprehensive review covered those approaches and offered a procedure for cloning of recombinant xylanase enzymes with thermostability and alkaline stability [14]. There are many computational approaches for predicting the enzyme activity from its tertiary structure [15,16], but the prediction of the activity of an enzyme based on its sequence is not a straightforward task. The members of a specific enzyme family, e.g., xylanases, have very similar sequences with high sequence identities, but very different activity levels in similar conditions. This property makes it very hard to predict the activity only from the sequence. The purpose of the proposed computational method is to predict the activity of enzymes from xylanase family based on limited experimental studies.

Different *Bacillus subtilis* strains capable of xylanase production have been hitherto isolated from natural resources [17–23]. In this study, 41 strains of *Bacillus subtilis* were isolated from gardens and farms based on their ability to produce xylanase enzyme.

For these strains, the xylanase activity determination experiment was done. Using trained computational models, the halo zone diameter in screening plates as well as enzyme activities, could be predicted based on Pseudo Amino Acid Composition (PseAAC) features that were extracted from xylanase amino acid sequences. This makes it possible to predict the bacterial halo diameter and enzyme activity in specified condition without doing screening and activity measurement experiments.

The main reason for choosing PseAAC feature vectors as representative of xylanase enzymes in activity prediction task was the fact that PseAAC features have been vastly used in computational biology for prediction of different properties of proteins and nucleic acid sequences since 2001 [24–58]. Some of its recent applications are related to RNA and DNA sequence analysis fields. Pseudo k-tuple nucleotide compositions (PseKNC) were exploited to identify enhancers and their strength in a two-layer architecture and since 2015 it has been accessible via iEnhancer-2L web server [34]. In 2016, two ensemble learning methods were introduced. The iDHS-EL is a web server for identifying DNase I hypersensitive sites which fuses three different modes of pseudo nucleotide composition [33]. Also, the iRSpot-EL fuses different modes of PseKNC plus mode of dinucleotide-based auto-cross covariance for identifying DNA recombination spots [59]. One of the most recent studies in 2017 introduces 2L-piRNA, a two-layer ensemble classification system, for identifying Piwi-Interacting RNAs and their function using PseKNC [60].

Among the important factors in industrial processes are pH and temperature on which chemical and enzymatic stability depend. Therefore, choosing the right enzyme to optimize catalyzing a specific reaction is not straightforward [61].

Many attempts have been made for engineering thermostable microbial xylanases for optimizing their activity in industrial processes through advanced biotechnological approaches including enzyme immobilization methods, gene editing and docking [62–64]. Despite the above mentioned studies, there is still no computational framework to predict the enzyme specific activity in the specified condition. The proposed approach can facilitate this complex process using statistical learning methods. Moreover, this method can be extensively used for screening the activities of enzymes extracted from metagenomic data.

## Materials and methods

### Experimental data

#### Bacterial strains and culture condition

About 90 *Bacillus subtilis* isolates were obtained from Microbial Culture Collection established in the Agricultural Biotechnology Research Institute of Iran (ABRII). The strains were grown in NBY medium (Nutrient Broth: 8g / L, K2HPO4: 1g / L, Yeast Extract: 1g / L, KH2PO4: 0.25g / L, Glucose: 2g / l, MgSO4 (1M): 1ml / L and Agar: 18g / L) and incubated at 28°C for 48 h.

#### Screening of xylanase producing bacterial isolates

Bacterial isolates were grown on XC agar medium containing 10 g/L oat-spelt xylan, 5 g/L peptone, 1 g/L yeast extract, 4 g/L K2HPO4, 1 g/L MgSO4.7H2O, 0.2 g/L KCl, 0.02 g/L FeSO4.7H2O, agar 15 g/L, pH 7.0. The plates were incubated at 28°C for 48h. Xylanse producing bacteria exhibited a clear zone around their colony as a qualitative index for xylanase productivity potential.

#### Enzyme production

For crude enzyme production, 200μl of overnight-grown bacterial culture in nutrient broth (OD600nm = 0.5) was transferred into 10 ml enzyme medium and shaked at 28°C for 48h. The enzyme medium contained xylan: 12 g/L, Meat Extract: 3 g/L, Yeast Extract: 4 g/L, CaCl2.H2O: 0.5 g/L, MgSO4.7H2O: 0.3 g/L and K2HPO4: 1 g/L and pH was adjusted to 7.0. The fermented culture medium was centrifuged at 10,000 rpm for 10 min at 4°C and the supernatant was stored at -20°C for xylanase assay.

#### Xylanase assay

Xylanase activity was assayed by measuring the formation of reducing sugar by the dinitrosalicylic acid (DNS) method [65]. The reaction mixture containing 100 μl of crude enzyme and 300 μl 1%soluble xylan(sigma) in 50 mM phosphate or citrate buffer at desired pH. After 20 min, the 600 μl DNS reagent was added to the mixture and boiled at 100°C for 15 min. The xylanase was assayed at three different conditions including temperature = 60°C and pH = 4.6, temperature = 26°C and pH = 4.6 and temperature = 26°C and pH = 6.9. Absorbance was measured at 540 nm against a reagent blank. A series of xylose dilutions were used as standards to calculate the quantity of reduced sugar. One unit (U) of xylanase activity was defined as the amount of enzyme needed to generate 1 μmol of reduced sugar per minute under the assay conditions.

#### Collected dataset

The xylanases were extracted from 41 different strains of *Bacillus subtilis*. Their GenBank accession numbers and the associated strain codes are demonstrated in Table 1. Also, Their amino acid sequences are provided in supplementary S2 Table. The diameter of bacterial halos was measured. Among 41 xylanases mentioned in Table 1, 28 enzymes were selected for determining their activities in different conditions of temperature and pH.

**Table 1:** 41 different xylanase enzymes were selected for experimental and computational studies. The GenBank Accession No., and its relevant strain code, for each sequence are included. The diameter of halos produced in the screening plates is also included for enzymes with high and medium halos surface. The last three columns shows the activities measured for 28 selected xylanase enzymes in three different pH and temperature conditions.

					*Activity (IU ml*^*-1*^*)*		
*No*.	*GenBank Accession No*.	*Strain*	*Halo zone diameter (mm)*	*Real Class*	*pH = 4* *T = 60°C*	*pH = 4* *T = 26°C*	*pH = 6* *T = 26°C*
**1**	AGO02713	a14h	4.6	M	400	280	320
**2**	AGO02715	d16d	5.7	H	295	80	300
**3**	AGO02724	d19d	5	M	490	360	370
**4**	AGO63347	d3d	5	M	60	120	150
**5**	AGO63342	h11h	5	M	590	380	205
**6**	AGO02730	h13f	4.2	M	170	200	150
**7**	AGS78259	h13h	4.1	M	490	370	320
**8**	AGO63354	h14d	6.5	H	740	120	170
**9**	AGO63351	h14h	5	M	810	130	330
**10**	AGO63345	h16h	4.8	M	330	205	150
**11**	AGO63356	k2b	7	H	670	230	250
**12**	AGO02722	k32l	5	M	280	290	150
**13**	AGO02727	k33l	5	M	440	660	330
**14**	AGO63344	k36p88	5	M	400	230	180
**15**	AGO63350	k40b	6	H	610	320	200
**16**	AGO02714	k43l	5	M	220	180	50
**17**	AGO02728	k46b	4	M	510	60	210
**18**	AGO02725	s6a	5.8	H	710	420	40
**19**	AGO02721	s7e	6.5	H	890	420	780
**20**	AGO02726	S7h	5	M	370	350	280
**21**	AGO97103	t27b	4.3	M	530	280	210
**22**	AGO02717	t28d	5	M	525	170	150
**23**	AGO63355	t31d	4	M	5	40	50
**24**	AGO63353	t34b	4.5	M	210	0	120
**25**	AGO02716	t37a	8	H	670	280	590
**26**	AGO02718	t41a	5	H	505	310	390
**27**	AGO02729	W	4.5	M	410	110	125
**28**	AGO63357	Y	4.5	M	690	390	260
**29**	AGO02712	b16b	3	L			
**30**	AGO02719	s2f	2.9	L			
**31**	AGO02720	s2h	2.5	L			
**32**	AGO02732	a10d	3.5	M			
**33**	AGO02723	d3b	2.5	L			
**34**	AGO02731	b5d	3	L			
**35**	AGO02733	s3d	2	L			
**36**	AGO63358	b9h	2.7	L			
**37**	AGO63360	s5d	3	L			
**38**	AGO97104	h13d	3	L			
**39**	AGO02734	S1d	3.8	M			
**40**	AGO63359	k27k88	3.5	M			
**41**	AGO63349	b11h	3.5	M			

All cloned xylanase gene sequences belongs to the CAZy GH family 11 according to the Expert Protein Analysis System (ExPASy) PROSITE.

The xylanases in rows 38–41 are excluded because they showed very low activities in all three different conditions of temperature and pH. By experimentally determining the activities for 28 sequences in three conditions, they were used as the material for building and validating a regression model to predict the activity of the xylanase enzymes. The model was validated using stratified k-fold cross validation and jackknife methods.

### Computational analysis

#### Feature extraction

From the machine learning perspective and, for computational prediction of enzymes activity solely from the sequence, the first step is extracting informative feature vectors based on the amino acid sequence of enzymes. These vectors are considered as the identity profile for each member of the enzyme family. It is expected that using these discriminative profiles and employing powerful computational methods, the activity level of novel enzyme sequences can be estimated. For learning the predictive model for a specific enzyme family, it is necessary to experimentally obtain the enzymatic activities for a limited number of enzyme sequences as training data. One of the well-known sequence based features which has been used in many computational tasks is the amphiphilic Pseudo Amino Acid Composition (PseAAC).

The concept of PseAAC was proposed by Chou [25]. Since then, the concept of PseAAC has penetrated into almost all the fields of computational proteomics [26–30,58]. Encouraged by the successes of introducing the PseAAC approach into computational proteomics, a novel feature vector, called ‘pseudo K-tuple nucleotide composition’(PseKNC) [31,32], was developed to represent DNA sequence samples to improve the quality of predicting the elements [33–37,39,40,57,66]. Some soft packages or web servers were established to produce the PseKNC [41–43]. The Pse-in-One is a web server with the ability of generating totally 28 different modes of pseudo components for DNA, RNA, and protein sequences [43]. Also, the Pse-Analysis is a Python package freely accessible at http://bioinformatics.hitsz.edu.cn/Pse-Analysis/ [67]. It provides an automated pipeline including feature extraction from samples and parameter selection, training and validating the model, and evaluating the quality of prediction.

The method of calculating the PseAAC vectors from the amino acid sequence is described in details in [68] and [24].

Suppose an enzyme *E*, with a sequence of *L* amino acid residues:

$$E=E_{1}E_{2}E_{3}\ldots E_{L}$$ (1)

In which *E*_*i*_ (*i* = 1,2,…,*L*) denotes the residue at chain position *i*. The hydrophobicity or hydrophilicity of amino acids plays important role in enzyme structure and hence its function [44]. Therefore, these indices are strong candidates to reflect the function and activity of enzyme sequences. The following equations reflect the sequence order effect of an enzyme in its activity and functionality:

$$\{\begin{array}{c} \tau_{2k-m}=\frac{1}{L-k}\sum_{i=1}^{L-k}H_{i,i+k}^{m}\\ k=1,2,\ldots ,\lambda ;\;\lambda <L\\ m=0\;or\;1 \end{array}$$ (2)

In above equations, *τ*_2*k*−1_ and *τ*_2*k*_ are called the *k*^*th*^-tier correlation factors and $H_{i,i+k}^{1}$ and $H_{i,i+k}^{2}$ are the hydrophobicity (*m* = 0) and hydrophilicity (*m* = 1) correlation functions respectively.

**Fig 1:**
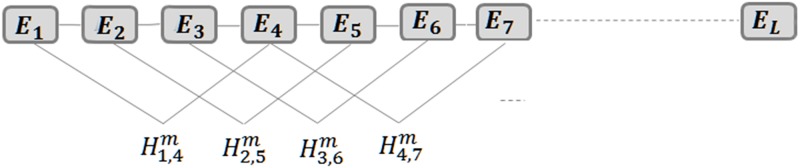
The amphiphilic coupling between all the third most contiguous amino acids. The values 0 and 1 for *m* represents the correlations via hydrophobicity and hydrophilicity indices.

*τ*_2−1_ and *τ*_2*k*_ reflect the sequence–order amphiphilic correlation between all the *k*^*th*^ most contiguous residues along the enzyme sequence. For example, *τ*_5_ and *τ*_6_ are the 3rd-tier (*k* = 3) correlation factors that shows the sequence-order correlation between all the 3^rd^ most contiguous residues in the sequence (Fig 1).

We used the PseAAC, a web server which is designed to generate PseAAC features from protein sequences [46] (http://www.csbio.sjtu.edu.cn/bioinf/PseAAC/).

For each enzyme sample E, we have an augmented vector to represent it:

$$E=\left[\begin{array}{c} e_{1}\\ \vdots \\ e_{20}\\ e_{21}\\ \vdots \\ e_{20+\lambda }\\ e_{20+\lambda +1}\\ \vdots \\ e_{20+2\lambda } \end{array}\right]$$ (3)

The elements of *E* are defined follows:

$$e_{k}=\{\begin{array}{c} \frac{f_{k}}{\sum_{i=1}^{20}f_{i}+w\sum_{j=1}^{2\lambda }\tau_{j}};\left(1\leq k\leq 20\right)\\ \frac{w\tau_{k}}{\sum_{i=1}^{20}f_{i}+w\sum_{j=1}^{2\lambda }\tau_{j}};\left(21\leq k\leq 20+2\lambda \right) \end{array}$$ (4)

Different values for *λ* produce different feature vectors for enzymes. In this study, due to slightly better performance, *λ* = 7 is used for generating feature vectors.

The feature values extracted from studied xylanase sequences are tabulated in supplementary S1 Table.

#### Building classification models

For constructing and learning a model to predict the bacterial halo diameters without the need for experimental works, 41 collected strains were cultured in selective environments and the halo diameters were measured. These results and PseAAC features obtained from respective xylanase sequence were used as the training and testing datasets for classifiers.

The Naïve Bayes, SVM (Support Vector Machine), K-Nearest Neighbor (KNN) (with K = 1) and random forest classifiers were used classify the diameter of halos. For KNN classifier with uniform weights and Euclidean distance, different values for K (from 1 to 15) were considered and for K = 1 the best performance was obtained.

SVM classifier with linear kernel, and random forest classifier with 30 trees were employed. The target class has been selected based on the majority vote from the individually trained trees in the forest. These classifiers were also used in many previous studies including virion protein prediction [48] DNA/RNA modified site identification [47,69], membrane transporter prediction [49] and the origin of replication prediction [39].

To compare the methods, the Area Under Curve (AUC) of ROC curve, Classification Accuracy (CA), F1, precision and recall measures obtained from classification methods were used.

#### Building regression models

The statistical process of estimating the relationships between a dependent variable and one or more independent variables is called regression. In fact, the conditional expectation of dependent variable is estimated given the independent variables, or predictors. In this study, the enzyme activity in a fixed pH and temperature was estimated based on PseAAC features.

We used different regression methods to determine the activity of xylanases with slightly different sequences using PseAAC vectors in three different pH-temperature conditions.

SVM, KNN and random forest regression algorithms were used to build a proper regression model for xylanase activities in different conditions. Also, boosting regression trees using Adaboost algorithm were examined. For SVM regressor, linear kernel was employed. For KNN, uniform weights and Euclidean distance were considered and different values for K (from 1 to 15) were tested. The best performance was achieved for k = 5. In Adaboost, 50 regression trees as the base regressor machine were fused. For each regression tree, at least two instances for each leaf and 5 instances for internal nodes with the maximum depth of 100 were considered. In the random forest regressor, 10 trees were generated and for each tree maximal tree depth and an unlimited number of considered features were used. The Mean Squared Error (MSE), Root Mean Square Error (RMSE), Mean Absolute Error (MAE) and R2 measures were calculated and used for comparing the results.

#### Implementation and validation of computational models

In this study, Orange was used for performing classification, regression and validation operations as an open source datamining and machine learning toolbox implemented in python [70]. Free access to python codes of Orange makes it possible to use it in the future development of web applications for similar studies.

K-fold cross-validation test, sub-sampling test, independent dataset test and jackknife cross-validation test are four kinds of strategies in statistical learning which have been widely used to examine the performance of a prediction model [71–73]. Because the jackknife test can achieve unique outcomes [74], it has been widely used in Bioinformatics [75–79]. However, the jackknife cross-validation is more time-consuming. In this study, the 10-fold cross-validation as well as the jackknife method were used to investigate the performance of the prediction models.

## Results

### Xylanase assay

Experimental screening resulted in isolation and identification of 41 isolates producing xylanase enzyme. Approximately, 28 xylanase producing strains (68%) had clear zones larger than 35 mm and selected for xylanase assay at three different conditions.

The halos with a diameter less than 3.5mm are assigned to class Low (L), between 3.5mm and 5.5mm are assigned to class Medium (M), and larger than 5.5mm are assigned to class High (H). Among these xylanases, 28 sequences related to strains with Halo Diameter (HD) greater than 3.5mm (from M or H classes) were selected for further analysis.

### Classification results

Applying Random Forest, Naïve Bayes, SVM, and KNN on 41 sequences listed in Table 1 for classifying the area of bacterial halos from respective expressed xylanase enzymes showed that the diameter and therefore the area of these halos could be classified with high accuracy in one of the three categories L, M, or H. Table 2 shows the results. The AUC, CA, F1, precision and recall measures for different models are reported in Table 2. The results were validated by 10-fold cross validation and jackknife methods.

**Table 2:** Results from three different classifiers.

*Model*	*AUC*		*CA*		*F1*		*Precision*		*Recall*	
	10 fold	Jackknife	10 fold	Jackknife	10 fold	Jackknife	10 fold	Jackknife	10 fold	Jackknife
***Random Forest***	1.00	1.00	1.00	1.00	1.00	1.00	1.00	1.00	1.00	1.00
***SVM***	0.857	0.854	0.854	0.829	0.856	0.831	0.861	0.834	0.854	0.829
***KNN (K = 1)***	0.964	0.910	0.951	0.902	0.951	0.900	0.954	0.901	0.951	0.902
***Naïve Bayes***	0.688	0.636	0.317	0.610	0.307	0.604	0.608	0.600	0.317	0.610

According to the results in Table 2, the Random Forest classifier outperformed other models.

### Regression results

**Fig 2:**
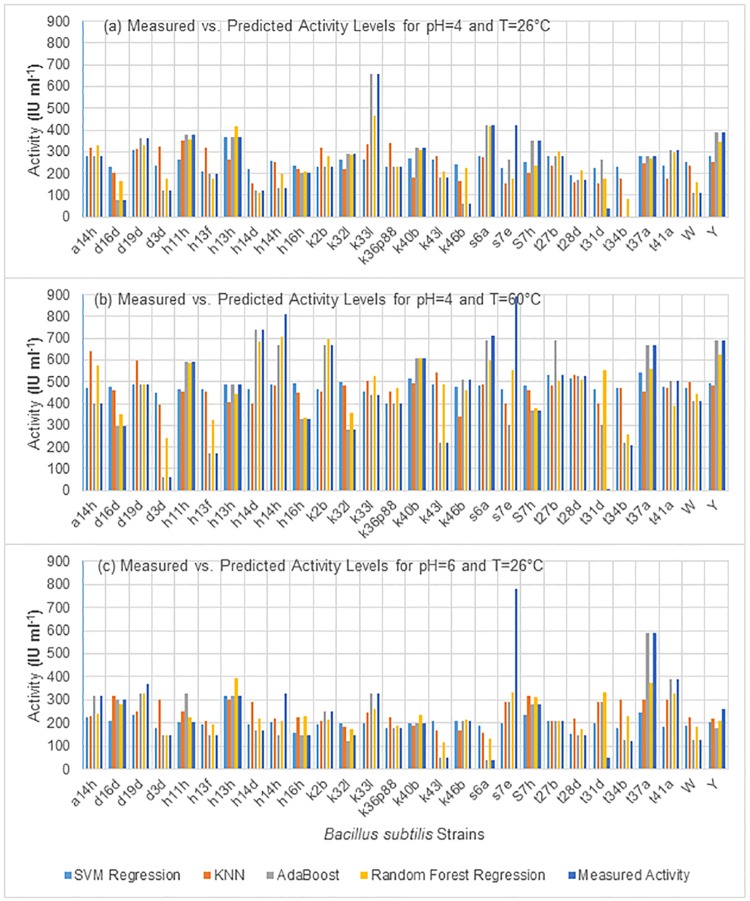
The activity of xylanase enzymes purified from different *Bacillus subtilis* strains vs. predicted activities by four computational models. The activities were determined in three different pH/temperature conditions. (a) pH = 4,T = 26°C (b) pH = 4,T = 60°C (c) pH = 6,T = 26°C.

Using SVM, KNN, Adaboost and random forest regression algorithms, predictive regression models were built for predicting the xylanase activities in three specific temperatures and pH conditions. The experimental data in Table 2 were used for tuning the parameters of these models. The results are demonstrated in Fig 2 and Table 3. Fig 2, shows the experimentally measured activities vs. predicted values by all four regression models for all 28 strains. Table 3 summarizes the performance measures for regression models. Based on the results, the SVM regressor showed the best performance. As it can be seen in Fig 2, except the s7e and t31d in all three conditions, and t34b in part (a) almost for all other strains the activity of produced enzyme has been accurately predicted by at least one of predictors. Despite the overall better performance of SVM, the Random Forest regressor showed better results.

**Table 3:** Performance measures resulted from four different regression models. The models were validated by stratified 10-fold cross validation and jackknife methods. The results are related to three different pH/Temperature conditions. (a) pH = 4,T = 26°C (b) pH = 4,T = 60°C (c) pH = 6,T = 26°C.

			Regression Models							
			*SVM*		*KNN (K = 5)*		*AdaBoost*		*Random Forest Regression*	
			*10-fold*	*Jackknife*	*10-fold*	*Jackknife*	*10-fold*	*Jackknife*	*10-fold*	*Jackknife*
**Assay Conditions**	pH = 4 T = 26°C	MSE	24529.116	22879.562	25600.679	26193.536	35004.789	39039.153	34166.515	29285.824
		RMSE	156.618	151.260	160.002	161.844	187.096	197.583	184.842	171.131
		MAE	126.693	122.028	135.536	135.893	147.176	162.334	139.415	134.655
		R2	-0.21	-0.128	-0.263	-0.292	-0.726	-0.925	-0.685	-0.444
	pH = 4 T = 60°C	MSE	55753.178	57096.944	72585.964	73541.250	123725.753	114543.750	78659.703	77522.968
		RMSE	236.121	238.950	269.418	271.185	351.747	338.443	280.463	278.429
		MAE	197.666	199.404	227.179	231.821	284.171	254.821	222.875	226.616
		R2	-0.192	-0.221	-0.552	-0.572	-1.646	-1.449	-0.682	-0.658
	pH = 6 T = 26°C	MSE	31188.785	29521.580	32759.964	32268.250	62414.683	59070.759	44906.883	44090.080
		RMSE	176.603	171.818	180.997	179.634	249.829	243.045	211.912	209.976
		MAE	130.15	126.440	134.179	132.321	182.262	160.089	149.517	159.520
		R2	-0.282	-0.213	-0.347	-0.326	-1.565	-1.428	-0.846	-0.812

## Discussion and conclusion

Understanding the properties of amino acid sequences from their primary structure is one of the main challenges in computational biology.

The rapid growth in the number of enzymes discovered from high-throughput sequencing generates a wealth of data. However, a major challenge is the functional assignment and activity prediction for many newly found enzymes with no or limited experimental data. The activity of an enzyme in a specified condition is a very important factor that can affect the rate of the underlying reaction.

It is worth noting that both enzyme molecular function prediction and enzyme specific activity prediction are important and challenging subjects which should not be confused with each other.

Enzyme molecular function prediction refers to identifying the biochemical reactions that an enzyme can catalyze and these functions are manually classified by the Enzyme Commission[80]. Several in-silico and experimental methods have been developed for this purpose, many of which are based on the identification of target substrates for the enzyme active site(s)[50].

However, for members of an enzyme family with similar molecular function, the level of catalytic specific activity can be very different for a given condition of temperature, pH and the presence of inhibitory factors. Establishing a computational framework for in-silico prediction of the specific activities for the members of an enzyme family only from their amino acid sequence, and for a given condition, is the main novelty of this research. Building a learning model with high generalization power needs adequate training samples. In this field, we need dozens of enzymes from the same family, with known amino acid sequence and precise specific activity values in the same pH and temperature to learn our regression model.

Despite many empirical studies which have been done to measure the activity of a variety of enzymes, due to the lack of enough proper training data for specific temperature/pH condition, very little has been done to build statistical learner models for activity prediction from sequence.

This research work is one of the primary steps to cover this deficiency. Due to the fact that screening the bacterial halos is a primary step for selecting proper strains, a method was proposed that can classify the magnitude of the diameter of bacterial halo zone by exploiting PseAAC features. In the xylanase selective medium, the halo diameter is highly correlated with the activity of the expressed xylanase from the corresponding strain. However, it is clear that the halo diameter is not the only function of the xylanase activity, but, many factors play a role in its formation. Therefore, the exact prediction of halo diameter only from xylanase sequence is impossible. Nevertheless, we showed that correct classification of halo diameter from the enzyme sequence in one of H, M, and L classes is logical and feasible. Therefore, we developed two learning models which help to obtain a relatively accurate estimation of activity for new xylanases and bacterial halos diameter for new strains without the need for new experiments. Finding a reliable in-silico prediction model for enzyme function and activity, may circumvent costly and time consuming experimental screening. We showed that the problem of enzyme activity prediction solely from its primary structure could be partially solved by regression machines. Adequate training data makes the regression results more reliable and informative. However, the accuracy and precision of predictors remain as serious concerns. The main reason is that choosing a model because of its performance based on limited training data, does not guarantee the correct prediction of future observations, also known as the generalization power of predictor. Cross-validation is a technique for evaluating predictive models and assesses how the performance of a learning model will be generalized to independent and unseen datasets. Our proposed models were validated using stratified cross-validation and the jackknife techniques.

Although PseAAC features have been used in many prediction tasks in computational biology, to the best of our knowledge, its usage for determining the activity of enzymes in specific conditions has not been reported yet. Further efforts are required to develop similar computational models for enzyme activity prediction based on the other bio-physicochemical and evolutionary features that can be extracted from the amino acid sequence of enzymes. The features obtained from PSSM (Position Specific Scoring Matrix), hydrophobicity, polarity, polarizability, and many others are among such sources of information about enzyme activity.

In this work we used a feature vector with 34 elements. Using other information sources such as the above mentioned features, can heavily increase the feature vector dimension. In machine learning tasks, high dimension feature will maybe result in three problems: one is over-fitting which results in low generalization ability of prediction model; another is information redundancy or noise which results in bad prediction accuracy; the other is dimension disaster which results in a handicap for the computation. Using feature selection techniques to optimize feature set can not only economize the time for computation, but also build robust prediction model. In fact, many techniques such as principal component analysis (PCA) [53], minimal-redundancy-maximal-relevance (mRMR) [54], analysis of variance (ANOVA) [55], F-score algorithm [40], binomial distribution [56] have been proposed and used in sequence analysis and prediction. Thus, feature selection in the future works hopefully can improve prediction results.

As main achievement, the proposed methodology can be used for any family of enzymes, with exploiting any kind of regression machine and any sequence based feature vectors other than those discussed in this work. The only limitation is the availability of sufficient training data for specified temperature and pH condition.

No single general computational approach alone is likely to be a perfect solution for the problem of predicting the activity of homologous enzymes from different families [50]. However, it is possible and plausible for a specific family of enzymes to determine the activity of some members based on the determined activities of the others. In the current state, the lack of computational tools with the capability of enzyme activity prediction is tangible in both scientific studies and industrial applications. Considering the diversity of enzyme families and large number of members in each family, it seems very difficult to design a general purpose machine that can accurately predict enzyme activity in different pH and temperature conditions only from sequence based data. It is more practical to design and implement a special purpose predictor machine for each family of enzymes. These machines can be trained based on experimental activity measurements and evaluated with proper testing datasets. One of the main applications of predictive models similar to those introduced in this work is to select new suitable candidate enzymes with superior activities from huge metagenome data. It is almost impossible to select good targets without automated activity prediction tools. Since user-friendly and publicly accessible web-servers represent the future direction for developing practically more useful models [38,81–86], more efforts will be made in the future work to provide a web-server for the method presented in this paper.

## Supporting information

S1 Table The PseAAC feature values. Features extracted from 41 studied xylanase amino acid sequences. *λ* = 7 was used for generating features.
(XLSX)

S2 Table Studied xylanase sequences. The amino acid sequences of 41 different xylanase enzymes and their GenBank Accession No. are provided.
(XLSX)
